# Transcriptome-Wide N6-Methyladenosine Methylome Alteration in the Rat Spinal Cord After Acute Traumatic Spinal Cord Injury

**DOI:** 10.3389/fnins.2022.848119

**Published:** 2022-05-30

**Authors:** Jiangtao Yu, Haihua Chen, Haoli Ma, Zhongxiang Zhang, Xiaolu Zhu, Pengcheng Wang, Ruining Liu, Xiaoqing Jin, Yan Zhao

**Affiliations:** ^1^Emergency Center, Zhongnan Hospital of Wuhan University, Wuhan, China; ^2^Hubei Clinical Research Center for Emergency and Resuscitation, Zhongnan Hospital of Wuhan University, Wuhan, China; ^3^Department of Biological Repositories, Zhongnan Hospital of Wuhan University, Wuhan, China

**Keywords:** traumatic spinal cord injury, mRNA, methylation, epigenetic, MeRIP-Seq, m6A (N6-methyladenosine)

## Abstract

Recent studies showed that RNA N6-methyladenosine (m6A) plays an important role in neurological diseases. We used methylated RNA immunoprecipitation sequencing (MeRIP-Seq) technology to generate the m6A modification map after traumatic spinal cord injury (TSCI). A total of 2,609 differential m6A peaks were identified after TSCI. Our RNA sequencing results after TSCI showed 4,206 genes with significantly altered expression. Cross-link analysis of m6A sequencing results and RNA sequencing results showed that 141 hyper-methylated genes were upregulated, 53 hyper-methylated genes were downregulated, 57 hypo-methylated genes were upregulated, and 197 hypo-methylated genes were downregulated. Among these, the important inflammatory response factor *Tlr4* and the important member of the neurotrophin family *Ngf* were both upregulated and hyper-methylated after TSCI. This study provides that in the future, the epigenetic modifications of the genes could be used as an indicator of TSCI.

## Background

Acute traumatic spinal cord injury (TSCI) is a serious clinical traumatic disease that often leads to severe or permanent disability. According to the statistics of The National Spinal Cord Injury Statistical Center of America, the incidence of TSCI in the United States in 2010 was approximately 40 cases per year/1,000,000 persons, or about 12,400 cases per year (Devivo, 2012). The primary causes of TSCI are traffic accidents, falls, or sports-related injury (Chen et al., 2016). The cumulative direct medical expenses over a patient’s lifetime range from $500,000 to $2 million, a heavy burden on families and society (de la Torre, 1976; Sekhon and Fehlings, 1976; Albin and White, 1987).

Since the occurrence of TSCI cannot be prevented, the development of treatment options has become crucial (Falci et al., 2009; Ahuja et al., 2017; Hachem et al., 2017). However, there is still a lack of effective pharmacotherapies due to our incomplete understanding of the physiological and pathological mechanisms on secondary injuries after TSCI. As currently known, the pathogenesis of TSCI can be divided into primary and secondary injuries (McDonald and Sadowsky, 2002; Dietz and Fouad, 2014; Ahuja et al., 2017). The primary injury immediately causes mechanical rupture and dislocation of the spine, resulting in compression or transection of the spinal cord. The secondary injury cascade then occurs within minutes of the primary injury and lasts for months or more than half a year (Pineau and Lacroix, 2007). The mechanism of secondary injury is complex and is not yet fully understood. Possible mechanisms include ischemia, hypoxia, inflammation, edema, excitatory toxicity, ion steady state imbalance, and apoptosis (Schanne et al., 1979; Kwon et al., 2004; Brockie et al., 2021). Moreover, current research on the treatment of spinal cord injury mainly focuses on treatments such as neuroprotective therapy, nerve regeneration therapy, cell transplantation, neuromodulation, and robotics (Hall and Braughler, 1982; Hurlbert et al., 2015). Unfortunately, there is still a lack of recognized clinically approved therapeutic drugs for the treatment of the subacute phase of TSCI (Kwon et al., 2004).

Post-transcriptional modification has become recognized as an important regulatory factor in various physiological and pathological processes and has attracted increasing attention in biological research (Fu et al., 2014; Meyer and Jaffrey, 2014). The N6-methyladenosine (m6A) modification is the most abundant mRNA modification (Geng et al., 2020). A methyltransferase (Writer), a demethylase (Eraser), and a reader protein (Reader) are all required for the regulation of the m6A of target genes (Zhuang et al., 2019; Qin et al., 2020; Zhang H. et al., 2020; Ye et al., 2021). The level of modification affects the stability and translation efficiency of a target gene (Ma et al., 2019). In recent years, research has focused on m6A modification in neurological diseases. It has been reported that m6A modification alleviates ischemic stroke by promoting Akt phosphorylation by disrupting the stability of *Pten* mRNA (Zhang Z. et al., 2020). During cerebral ischemia and reperfusion, ALKBH5 and FTO selectively demethylate *Bcl2*, preventing degradation of *Bcl2* transcripts. These results highlight that m6A modification plays an important role in neurological diseases (Du et al., 2020; Xu H. et al., 2020; Xu K. et al., 2020). Downregulation of m6A mRNA methylation was found to be involved in Parkinson’s disease (Chen et al., 2019). Oxygen glucose deprivation leads to an increase in the level of m6A modification of Lnc-D63785, which leads to the accumulation of miR-422a and induces neuronal cell apoptosis (Xu S. et al., 2020). In the early stage of acute ischemic stroke, the level of METTL3 increases, which promotes an increase in the methylation and expression level of miR-355, thereby promoting stress granule formation (Si et al., 2020).

However, the study of m6A modification after TSCI in mammals has not been reported. In this study, we obtained TSCI and sham spinal cord tissues for Methylated RNA Immunoprecipitation Sequencing (MeRIP-Seq). Moreover, we investigated the function of mRNAs with significantly changed peaks by gene ontology (GO) and Kyoto Encyclopedia of Genes and Genomes (KEGG) analyses. Subsequently, a conjoint analysis was performed to reveal the significantly changed mRNAs with significantly altered enrichments that may play a vital role in the epigenetic modification of TSCI. Thus, we provide data for the exploration of TSCI pathological mechanisms and the development of effective treatment drugs.

## Materials and Methods

### Animals

All processes in this study followed the guidelines of the National Institutes of Health Laboratory Animal Care and Use Guidelines, and the study was approved by the Institutional Animal Care and Use Committee of Wuhan University (IACUC: ZN2021119). The male Sprague–Dawley rats (7 weeks) used in the study were purchased from Vital River Laboratory Animal Technology Co. Ltd. (Vital River, Beijing, China) and housed at the Animal Experiment Center of Zhongnan Hospital at Wuhan University. All the rats were placed in a regular environment with a 12 h light/dark cycle for 1 week before experiments with a weight ranging from 250 to 300 g.

### Animal Model of Traumatic Spinal Cord Injury

Rats were anesthetized with an intraperitoneal injection of 5% pentobarbital (50 mg/kg) and placed in the prone position. Next, after skin preparation and positioning, we performed laminectomy to expose the T10 spinal cord (Lee et al., 2014). An impact device (6800 II; RWD Life Science Corp, Shenzhen, China) was used to create an TSCI with an energy of 25 gcm. Wagging tail reflection, body trembling, and lower limb retraction were observed after impact. After surgery, the rats were injected with antibiotics for 3 days and were helped to empty their bladder until bladder function was restored.

### Hematoxylin and Eosin Staining

The T7–T12 spinal cords of rats were dissected 14 days after trauma. All the spinal cords were fixed in a 4% paraformaldehyde fix solution and then embedded in paraffin. Next, longitudinal serial sections (5 μm) from paraffin blocks were taken, and histopathological examination with Hematoxylin and Eosin (HE) staining was performed under a light microscope (Olympus BX53).

### Basso–Beattie–Bresnahanlocomotor Rating Scale Measurements

Basso–Beattie–Bresnahan (BBB) motor function scoring (Basso et al., 1995) was performed 1, 7, and 14 days post surgery to evaluate hind limb motor function. Each test was double-blinded by three professionally trained research graduate students in the laboratory. Before the experiment, the motor function of each rat was determined to be normal. For each test, the rats were placed in an open basin, and a researcher gently tapped the wall of the basin, making it crawl, and observing the hip, knee, ankle joint walking, trunk movement, and coordination. The total score was 21 points, and the higher the score, the more perfect the motor function.

### Inclined Plane Test

An inclined plane experiment (Kazanci et al., 2017) was carried out at 1, 7, and 14 days after the operation to evaluate the balance ability of each rat. A rat was placed on a plane equipped with an indicator indicating the inclination of the plane. Then, the plane was tilted at a speed of 2° per second, and the angle at which the rat fell from the plane was recorded as the falling degree. Each test was repeated three times, and the average value was recorded.

### Quantitative Reverse Transcription Polymerase Chain Reaction

The T7–T12 spinal cords of rats were dissected 14 days after trauma and immediately frozen in liquid nitrogen and stored at −80°C. Total RNA was extracted and purified using the RNAeasy™ Animal RNA Isolation Kit with Spin Columns (R0024). Next, we analyzed the quality of RNA using enzyme-labeled instruments, and samples with A260/A280 ratios between 1.9 and 2.2 were used for further experiments. The primers used in quantitative reverse transcription polymerase chain reaction (qRT-PCR) were designed using the Primer3 website^1^ and listed in the additional file (Supplementary Table 1). Following the Biomarker One Step SYBR Green RT-qPCR Kit (RK02012, Biomarker Technologies) to construct the reaction system. After centrifugation and blending (D1008, SCILOGEX), qPCR reaction was performed using QuantStudio 1 real-time fluorescence quantitative PCR system (QuantStudio 1, Applied Biosystems). The original CT data were exported and input by Excel, and the data were calculated and analyzed by Delta CT method.

### Methylated RNA Immunoprecipitation-QPCR (MeRIP-QPCR)

Take out the Invitrogen Dynabeads Antibody Coupling Kit (14311D, LC BIO) and prepare magnetic beads as instructed after balancing at room temperature for half an hour. Blend magnetic beads with Synaptic System M6A-Antibody (202003, LC BIO). Add 2 g RNA, 100 ml m6A binding buffer and add ddH_2_O to supplement to 500 ml. The incorporation of these fragment RNA with m6A-immunomagnetic beads at room temperature for 1 h with vertical mixing machine for 7 cycles/min. Place EP tube on magnetic holder for at least 5 min. Then, the preheated 125 μl elution buffer was added, slowly blown and mixed, and incubated at 42°C for 5 min. After 5 min, blow again slowly and drain the supernatant to a new 1.5 ml EP tube. Repeat 3 times to elute the RNA fragments on the magnetic beads. The previous Elution buffer is mixed together and the EP tube is placed on ice. After 4 rounds of elution, the solution in the final EP tube was 500 ml. Use the RNeasy MinElute Cleanup Kit (74204, Qiagen) to purify RNA. Finally, a qPCR quantitative experiment was carried out with the Biomarker One Step SYBR Green RT-qPCR Kit (RK02012, Biomarker Technologies). Using Ct of control input, Ct of control IP, Ct of treatment group input, and Ct of control IP to calculate the changing trend of the peak.

### Methylated RNA Immunoprecipitation Sequencing

Using a mixture of three rats as a biological replicate, approximately 10 μg of double poly(A) selected RNA was generated from 400 μg of total RNA. Then, the poly(A) RNA was fragmented into 100 nucleotide long pieces using a thermocycler. m6A-methylated RNA fragments were then enriched by immunoprecipitation with an anti-m6A antibody (Synaptic Systems, Cat. No 202 003). Then, 100 ng of RNA (100 ng of input and 100 ng of post-m6A-IP positive fraction) was used to construct libraries utilizing the Illumina TrueSeq Stranded mRNA library prep kit. Using the KAPA library quantification kit (KAPABIOSYSTEMS Cat.NO KK4824) to quantify the library, we then submitted these libraries for high-throughput sequencing (Illumina Hiseq X10; Meng et al., 2014; Lan et al., 2019).

### Data Analysis

In order to obtain high-quality clean reads that could be used for subsequent analysis, Trimmomatic software (v0.36) was used to remove adapters from FASTQ data. We then used the default parameters of HISAT2 software (v2.1.0) to compare clean reads with the reference genome (vRnor_6.0) of Sprague–Dawley rats and retained only comparison reads for subsequent analysis. The Guitar R package (v1.1.1.18) was used to check the quality of the genome comparison result data. We then used MeTDiff software (v1.1.0), and an input sample was used as a control for peak detection. We also used MeTDiff software to detect significantly enriched peaks. Then, GO and KEGG enrichment analyses were performed using hypergeometric distribution testing. Motifs for peak sequences were generated using MEME-ChiP (v5.0.5). The input of MeRIP-seq was used as the RNA-seq data. DESeq2 software (V1.14.1) was used to standardize the count number of each sample gene (Basemean value was used to estimate the expression level), the multiple of difference was calculated, and NB (negative binomial distribution test) was used to test the significance of reads number. Finally, the differential protein coding genes were screened according to the difference multiple and difference significance test results.

The data analysis in the experiment was performed using SPSS software (V25.0, IBM, United States). Volcano maps and four-quadrant maps were drawn using Origin software (v9.8.0.200), and a peak map was drawn using IGV software (v2.3.5). To draw a protein interaction network, Cytoscape software (v3.7.2) was used.

## Results

### Basic Characteristics of N6-Methyladenosine Modification After Traumatic Spinal Cord Injury

**FIGURE 1:**
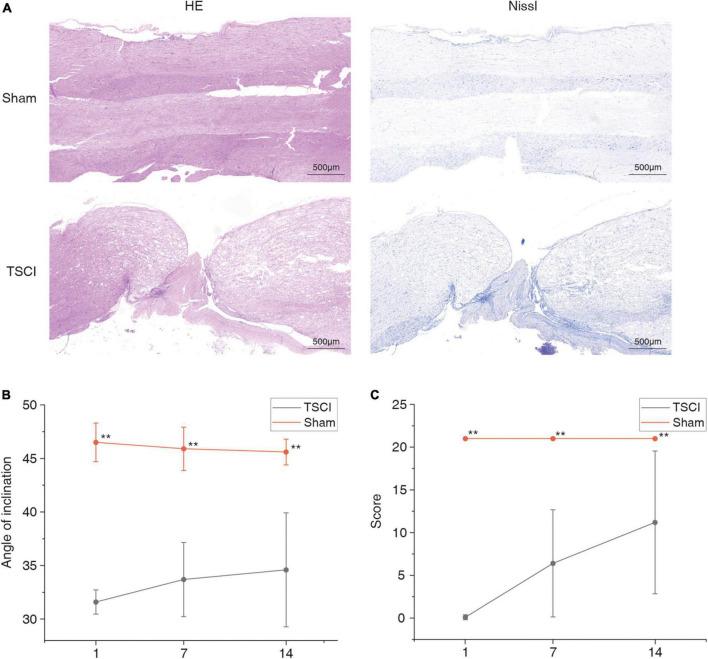
Pathological and behavioral changes after traumatic spinal cord injury. **(A)** HE staining and Nissl staining of sham operation group and traumatic spinal cord injury groups. **(B,C)** Basso–Beattie–Bresnahan (BBB) motor function and inclined plane experiment data for the sham and traumatic spinal cord injury groups. **p < 0.01.

**FIGURE 2:**
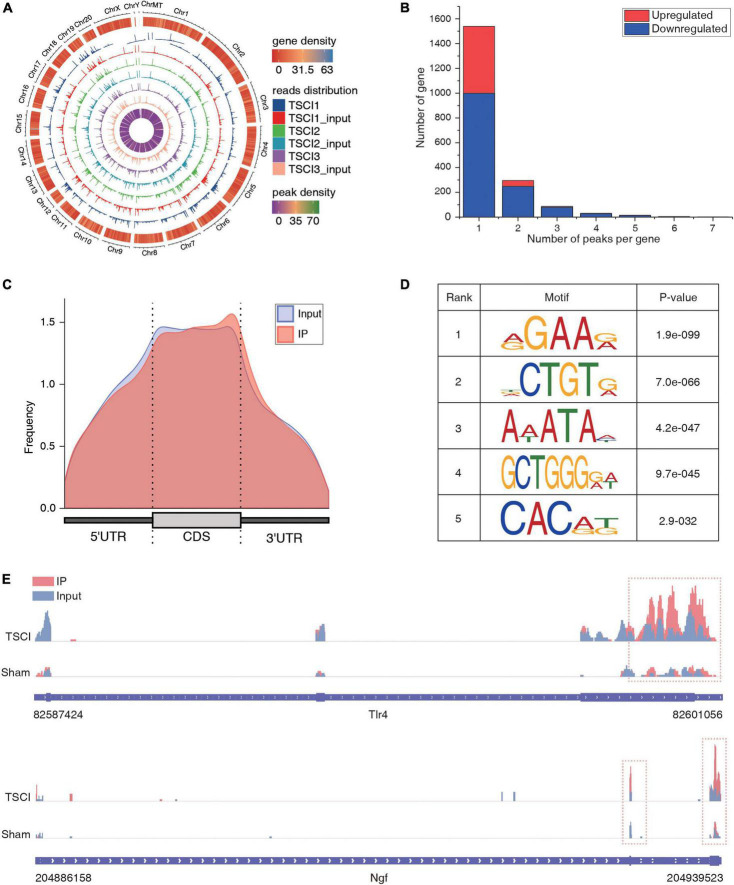
General feature of traumatic spinal cord injury, as determined by methylated RNA immunoprecipitation sequencing (MeRIP-Seq). **(A)** Distribution of reads across the genome. The circle map is in order from the outside to the inside. The outer circle is the genome length scale, the second outer circle is the gene density heat map of the species genome, the inner circle is the peak density heat map of the group of samples, and the rest of the circles are histograms of the distribution of reads of each sample on the genome. **(B)** Number of peaks per gene. **(C)** Read enrichment location on a gene. **(D)** Top five motifs based on *P* values. **(E)** Peak of *Tlr4* and *Ngf* in the TSCI and sham groups.

In order to evaluate the success of TSCI modeling, on the 14th day after surgery, samples were taken for HE and Nissl staining (Figure 1A). Compared with the control group, a large number of spinal cord nerve cells had died and tissues had atrophied after TSCI. Moreover, we performed BBB scoring and inclined plane experiments on 1, 7, and 14 days after surgery to verify the success of this model (Figures 1B,C). The BBB score and inclination angle of TSCI group were lower than those of sham group, indicating a significant decline in posttraumatic exercise ability. We used the tissues (T7–T12) obtained 14 days after TSCI to conduct transcriptome-wide m6A sequencing (m6A-seq) and RNA sequencing (RNA-seq). A total of 90.17 G of clean bases were obtained from six samples (each sample was mixed from two spinal cord tissues). The effective data volume of each sample was distributed from 5.37 G to 5.6 G, Q30 scores ranged from 90.32 to 95.3%, and the average GC content was 52.89% (Supplementary Table 2). By comparing the reads to the reference genome, the genome comparison of each sample was obtained, and the mapping rate was 87.23–93.32% (Supplementary Table 3). And reads are distributed throughout the genome (Figure 2A). A total of 22,701 peaks were identified in the TSCI group, and 24,108 peaks were identified in the sham group. There was only one peak for most genes, most of them were downregulated, and there were at most seven peaks for one gene (Figure 2B and Supplementary Table 4). Moreover, 43.35% of peaks were enriched in the 3′UTR, 5% of peaks were enriched in the 5′UTR, and 43.99% of peaks were enriched in the exon in the Sham group. Furthermore, 48.8% of peaks were enriched in the 3′UTR, 10.13% of peaks were enriched in the 5′UTR, and 41.07% of peaks were enriched in the exon in the TSCI group (Figure 2C). Our study identified the top five motifs to be “RGAAR,” “DCTGTR,” “AWATAH,” “GCTGGGRW,” and “CACRK” (Figure 2D). Figure 2E shows the peak of *Tlr4* (3′UTR) and *Ngf* (3′UTR) in the sham and SCI groups.

### Peak Alterations After Traumatic Spinal Cord Injury

**FIGURE 3:**
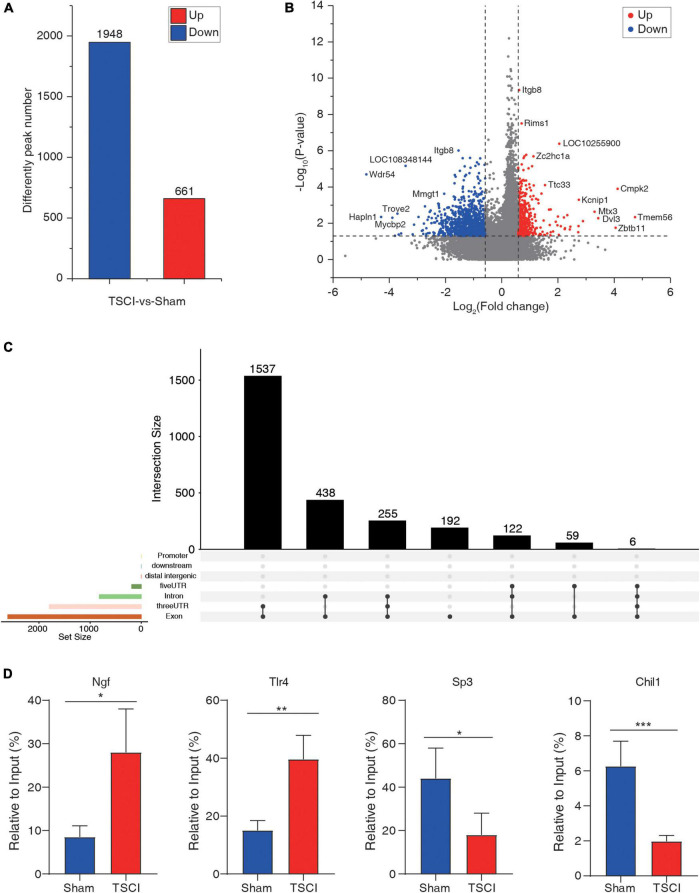
Difference peaks after traumatic spinal cord injury. **(A)** Number of upregulated and downregulated peaks after traumatic spinal cord injury. **(B)** Volcano map of significantly changed peaks after traumatic spinal cord injury. **(C)** Distribution of significantly changed peaks after traumatic spinal cord injury. **(D)** Methylated RNA Immunoprecipitation-qPCR (MeRIP-qPCR) of hyper-methylated (*Ngf, Tlr4*) and hypo-methylated (*Sp3, Chil1*) genes after TSCI (*p < 0.05; **p < 0.01; ***p < 0.001).

To investigate m6A methylation alteration after TSCI, differential m6A peaks were further analyzed. We identified an average of 22,701 peaks in the TSCI group and an average of 24,108 peaks in the sham group. The average length of peaks in the TSCI group was 4,696.27 bp, and that in the sham group was 4,476.93 bp. By comparing the peaks in the TSCI and sham groups, 1,948 hypo-methylated m6A peaks and 661 hyper-methylated m6A peaks were subsequently identified (*p* < 0.05, FC > 1.5; Figure 3A). A volcano chart showed the *p*-value and multiple changes of these differential peaks (Figure 3B). Moreover, the top 20 altered m6A peaks are listed in Table 1. The information on all the different genes is listed in Supplementary Table 5. We further investigated the location of significantly changed peaks and found that 1,537 differential peaks were located in the 3′UTR and exon regions, 438 differential peaks were located in the intron and exon regions, 255 differential peaks were located only in introns, and 192 differential peaks were located only in exons. A total of 122 differential peaks were located in the 5′UTR, intron, and exon regions, 59 differential peaks were located in the 5′UTR and exon regions, and 6 differential peaks were located in the 5′UTR, intron, 3′UTR, and exon regions (Figure 3C). In order to verify the accuracy of the sequencing results, we selected the hyper-methylated (*Ngf, Tlr4*) and hypo-methylated (*Sp3, Chil1*) genes after TSCI for MeRIP-qPCR, and the results showed good consistency (Figure 3D).

**TABLE 1:** The top 20 significantly changed N6-methyladenosine (m6A) methylation peak.

mRNA	Chromosome	Peak start	Peak end	Lg (*p*-value)	Log_2_ (fold-change)	Up/down
Nefm	Chr15	44856242	44857031	−12.7	0.62	Up
Prpf18	Chr17	77601963	77616775	−10.5	0.706	Up
LOC498453	Chr15	11650421	11650721	–8.99	2.05	Up
Lrrc32	Chr1	163454482	163455131	–8.24	0.868	Up
LOC108352579	Chr13	97445006	97445506	–8.13	1.13	Up
Bcl2l13	Chr4	153435810	153436655	–8.13	0.804	Up
Mrgprf	Chr1	218475189	218476030	–8.01	0.775	Up
Prpf38b	Chr2	211707281	211708683	–7.56	0.712	Up
Zc3h10	Chr7	2970111	2970778	–7.44	1.08	Up
Ttbk1	Chr9	16897276	16898021	–7.33	0.585	Up
Itgb8	Chr6	147089329	147091477	–8.53	–1.54	Down
Rims1	Chr9	28442553	28443003	–8.03	–1.13	Down
LOC102555900	Chr1	190673234	190674225	–7.99	–1.39	Down
Tom1l1	Chr10	78174045	78179473	–7.97	–0.768	Down
Gdap1	Chr5	1329061	1330197	–7.7	–0.88	Down
Zc2hc1a	Chr2	96482937	96488689	–7.58	–0.822	Down
Dlg2	Chr1	157270028	157271029	–7.54	–0.612	Down
Itgav	Chr3	71204463	71205510	–7.47	–0.936	Down
LOC108348144	ChrX	124513185	124516702	–7.46	–3.42	Down
Ppp1r12a	Chr7	51405566	51406659	–7.42	–1.32	Down

### Gene Ontology and Kyoto Encyclopedia of Genes and Genomes Analyses

**FIGURE 4:**
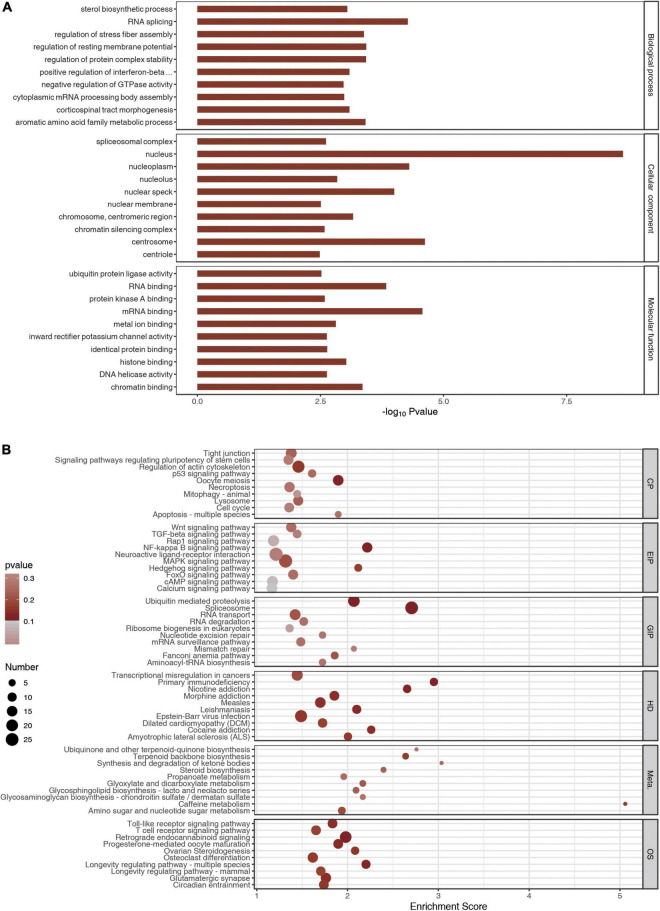
Gene Ontology (GO) and Kyoto Encyclopedia of Genes and Genomes analyses. **(A)** The top 10 GO terms of m6A peaks. **(B)** The top 10 meaningful and enriched pathways of m6A peaks.

Gene ontology and KEGG analyses were carried out using the genes with differential peaks to determine biological functions or pathways that these genes with differential peaks were mainly associated with. GO analysis showed that the differential peaks were related to the biological processes such as sterol biosynthetic process, RNA splicing, regulation of stress fiber assembly, and regulation of resting membrane potential. They were related to cellular components such as spliceosomal complex, nucleus, nucleoplasm, and nucleolus. Molecular functions such as ubiquitin–protein ligase activity, RNA binding, and protein kinase A binding were related to these significantly changed peaks (Figure 4A). KEGG analysis indicated that the differential peaks were concentrated mainly in cellular processes such as tight junctions, signaling pathway regulating pluripotency of stem cells, regulation of actin cytoskeleton, and p53 signaling pathway, and these were related to environmental information processing such as Wnt signaling pathway, TGF-beta signaling pathway, and Rap1 signaling pathway. There were also relations to genetic information processing such as ubiquitin-mediated proteolysis, spliceosome, RNA transport, and RNA degradation. These genes were also related to diseases such as transcriptional misregulation in cancers and to primary immunodeficiency and metabolism such as ubiquinone and other terpenoid–quinone biosynthesis and terpenoid backbone biosynthesis. In addition, these genes were related to organic systems such as Toll-like receptor signaling pathway, T-cell receptor signaling pathway, and retrograde endocannabinoid signaling pathway (Figure 4B).

### RNA Expression Profiles After Traumatic Spinal Cord Injury

**FIGURE 5:**
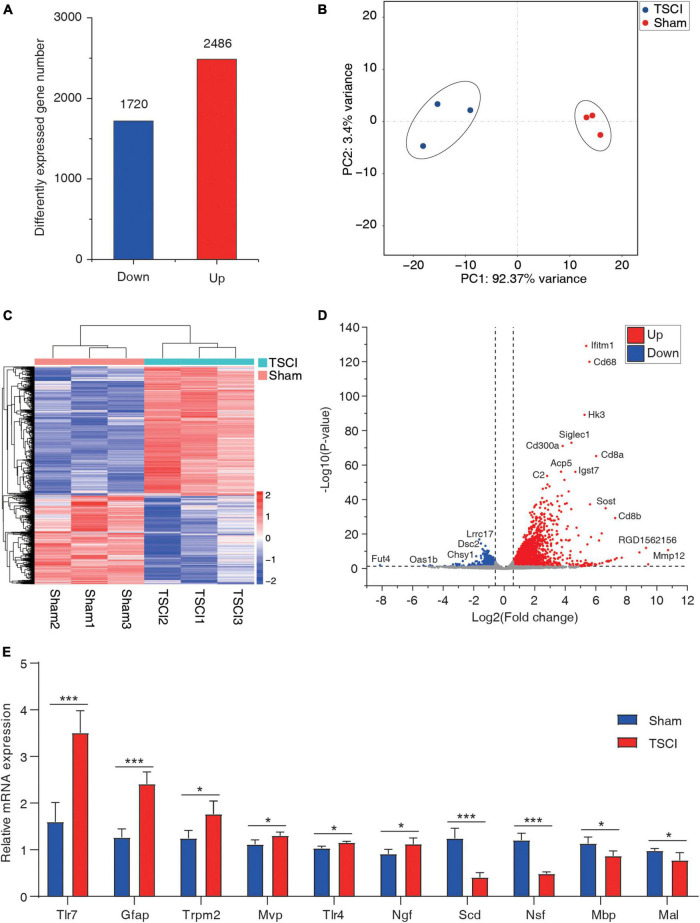
The basic information of differentially expressed mRNAs. **(A)** Number of upregulated and downregulated genes after traumatic spinal cord injury. **(B)** Principal component analysis of the sham and traumatic spinal cord injury groups. **(C)** Heat map of all samples. **(D)** Volcano plot of differentially expressed genes. **(E)** RT-PCR of upregulated genes (*Tlr7, Gfap, Trpm2, Mvp, Tlr4*, and *Ngf*) and downregulated genes (*Scd, Nsf, Mbp*, and *Mal*; *p < 0.05; ***p < 0.001).

By using the input library, this study generated a differential gene expression map of TSCI vs. sham, with a total of 1,720 downregulated genes and 2,486 upregulated genes (*p* < 0.05, FC > 1.5; Figure 5A). In the results of the principal component analysis, confidence ellipses separated the TSCI and sham groups, indicating that there was comparability between these two groups (Figure 5B). The heat map shows the relative expression levels of each sample in the TSCI and sham groups; samples in the same group showed similar patterns, and the two groups showed obvious different patterns (Figure 5C). The volcano graph shows the overall P value and foldchange of each difference peak (Figure 5D). Upregulated genes such as *Mmp12, Ifitm1*, and *Cd68* and downregulated genes such as *Fut4, Lrrc17*, and *Dsc2* are marked in this graph. Moreover, the top 20 differentially expressed genes are listed in Table 2. All genes with significant changes in expression after TSCI were listed in Supplementary Table 6. And upregulated genes (*Tlr7, Gfap, Trpm2, Mvp, Tlr4, Ngf*) and downregulated genes (*Scd, Nsf, Mbp, Mal*) were chosen to conduct the validation experiment. The qRT-PCR results showed good consistency with the sequencing result (Figure 5E).

**TABLE 2:** The top 20 significantly changed mRNA.

mRNA	Chromosome	Gene start (bp)	Gene end (bp)	*P*-value	Log2 (fold-change)	Up/Down
Ifitm1	1	213765825	213767841	4.3457E-134	5.368105879	Up
Cd68	10	56268726	56270605	1.1122E-124	5.583220003	Up
Hk3	17	10134726	10152976	1.16326E-93	5.26330004	Up
Siglec1	18	74403384	74417333	2.2165E-77	4.405105711	Up
Cd300a	10	103437978	103451170	2.3367E-75	3.825439556	Up
Cd8a	4	99217640	99243352	1.9388E-69	6.019460654	Up
Acp5	8	23142733	23149067	2.92002E-60	3.722110733	Up
Igsf7	10	104975782	104980853	3.6672E-60	4.663971798	Up
C2	10	105650866	105668579	9.86971E-58	2.809122238	Up
Plbd1	4	170564387	170620681	2.08627E-55	3.946813879	Up
Scd	1	264159966	264173061	1.5956E-19	−1.629678141	Down
Hmgcs1	2	52427351	52445082	4.87672E-17	−1.526587819	Down
Cyp51	4	27175564	27194018	1.55254E-15	−1.234384485	Down
Hsd17b7	13	88288116	88308019	1.30902E-13	−1.42223302	Down
Tm7sf2	1	221426017	221430340	9.7401E-13	−1.342201068	Down
Hmgcr	2	27480224	27500654	1.0878E-12	−1.115309377	Down
Aif1l	3	9237944	9262628	1.8537E-12	−1.015465487	Down
Fdft1	15	46339248	46367302	4.34558E-12	−1.239713017	Down
Sc5d	8	46525406	46537014	1.10621E-11	−1.053381747	Down
Sqle	7	99609929	99624803	1.80017E-11	−1.077083764	Down

### Crosslinking Analysis Results

**FIGURE 6:**
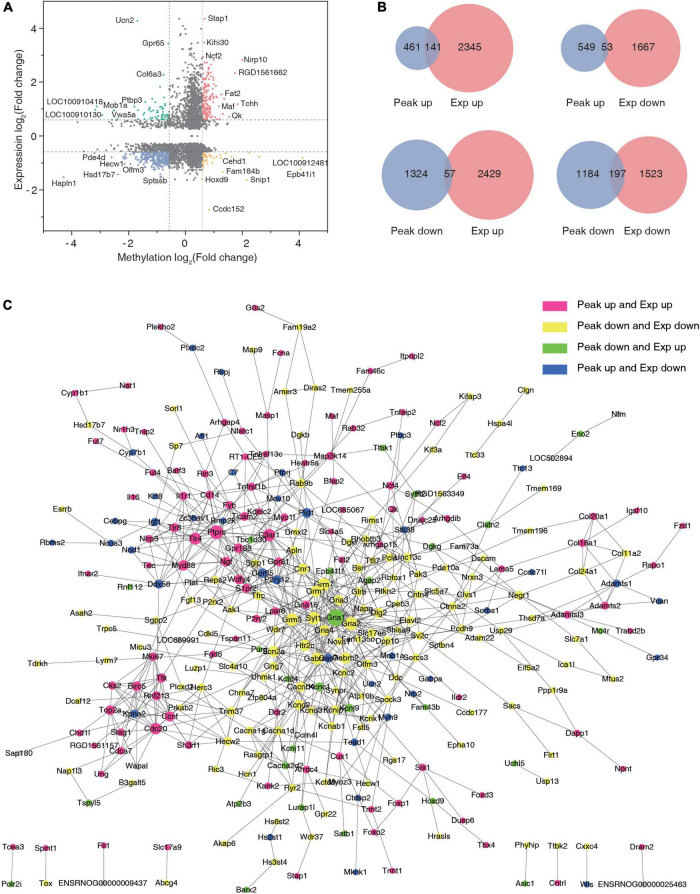
Conjoint analysis of N6-methyladenosine (m6A) methylation and mRNA expression after traumatic spinal cord injury. **(A)** The nine-quadrant graph of differentially expressed genes with significantly changed peaks. **(B)** Venn diagram of differentially expressed genes with significantly changed peaks. **(C)** Protein–protein interaction network.

Cross-linking analysis of differential peaks and differential expression of given genes was performed (Supplementary Table 7), and the four-quadrant graph shows the FC of differential peaks and differential RNAs (Figure 6A). A total of 141 hyper-methylated genes were upregulated, 53 hyper-methylated genes were downregulated, 57 hypo-methylated genes were upregulated, and 197 hypo-methylated genes were downregulated in the two groups (Figure 6B). Using the significantly changed genes with differentially methylated peaks, a protein interaction network diagram was created, wherein the different colors represent different gene expression and methylation trends. As shown in Figure 6C, the hyper-methylated genes *Ptprc, C3ar1, Myd88*, and *Cdc20* were upregulated after TSCI. The hypo-methylated genes *Grm3, Gria4*, and *Gria2* were downregulated after TSCI. The hyper-methylated genes *Brnp2K, Pld1*, and *Gpr65* were downregulated after TSCI. The hypo-methylated genes *Gria1, Agap2*, and *Kcnj9* were upregulated after TSCI.

## Discussion

An increasing number of studies have found that RNA m6A methylation plays an important role in central nervous system (CNS) injury disease and neurodegenerative diseases (Weng et al., 2018; Meng et al., 2019; Wang et al., 2021). However, there are currently no studies on how m6A is related to acute TSCI, partly because the methylation modification profile in TSCI is not yet clear. Using MeRIP-Seq, we have obtained a panoramic view of m6A methylation in the spinal cord after TSCI, which provides material for further in-depth study of m6A methylation in TSCI.

A total of 2,605 significantly changed peaks were identified in this study, among which 1,948 peaks were downregulated and 661 were upregulated. A summary of the peak sites indicated that the m6A peaks with different methylation in traumatic brain injury (TBI) were mainly distributed at the 3′UTR near the stop codon and at the 5′UTR near the start codon, which was consistent with the characteristics found in other sequencing studies. Peaks identified in a MeRIP-Seq conducted on the hippocampus of mice following TBI were enriched mainly in the coding sequencing near the stop codon (Wang et al., 2019). Moreover, research conducted on the cerebral cortex of rats following TBI showed a similar peak distribution pattern as that observed in our study (Yu et al., 2020).

We also conducted GO and KEGG analyses, and the most enriched pathway and the biological process had some similarities with other NCS injury disease datasets. The MeRIP-Seq conducted on mice hippocampus indicated that the significantly changed peaks were enriched in the regulation of actin cytoskeleton, rap1 signaling pathway, and MAPK signaling pathway, which were also the most enriched pathways in our research (Wang et al., 2019). A study conducted on C56BL/6J mice with focal ischemia revealed that significantly changed peaks were also enriched mostly in the p53, NF-KB, and MAPK signaling pathways, as was found in our study (Chen et al., 2018; Chokkalla et al., 2019; Ye et al., 2020). However, the m6A sequencing conducted on the neurodegenerative diseases showed different characteristics. In Alzheimer’s disease (AD), the significantly changed peaks were mostly enriched in synaptic growth at the neuromuscular junction, snRNA transcription, and the smoothened signaling pathway involved in dorsal/ventral neural tube patterning (Han et al., 2020). This phenomenon might be due to the fact that our sample was taken in the subacute phase of TSCI, while neuro degeneration mostly occurs during the chronic phase. Our results from cross-linking analysis showed that some TSCI-related genes were changed significantly and hypo- or hyper-methylated after TSCI. As is well known, all main CNS cell types express toll-like receptor 4 (*TLR4*). Moreover, our study found that *TLR4* was upregulated and hyper-methylated after TSCI. We found that TLR4 signaling is essential for maximal sparing and replacement of acute oligodendrocyte lineage cells after spinal cord injury (Church et al., 2016). Studies have revealed that FGF10 inhibits the activation and proliferation of microglia/macrophages by regulating the TLR4/NF-B pathway and inhibits the release of pro-inflammatory cytokines after TSCI (Chen et al., 2017). All these results indicate that *TLR4* plays important mediating roles in the pathological process of TSCI, and this has been well demonstrated. However, RNA methylation has been previously underrecognized, and its role remains to be clarified. As a member of the neurotrophin family, nerve growth factor (*Ngf*) has been demonstrated to modulate the survival of damaged neurons and facilitate axonal sprouting and regeneration (Iulita and Cuello, 2016; Indo, 2018; Hu et al., 2020). Moreover, our study found that *Ngf* was upregulated and hyper-methylated after TSCI. A study conducted in 2019 using a new delivery system to deliver *Ngf* to the spinal cord trauma site and the pathological and behavioral test results have confirmed that exogenous *Ngf* enables neural regeneration, tissue remodeling, and functional recovery in mice with TSCI. Also, as is well known, m6A can mediate mRNA expression by directly influencing its stability or by attracting readers to attach to mRNA. Some drugs [such as FB23-2(Yu et al., 2020)] have been developed to affect the enzymes related to m6A, and whether these drugs can be used to treat TSCI by influencing the *Ngf* level is an interesting question.

The study of m6A modification in CNS injury and neurodegenerative diseases has also unveiled some potential intervention targets (Mathiyalagan et al., 2019; Pan et al., 2021). FTO and METTL14, as important m6A mediation enzymes, have been found to be downregulated in rat cerebral cortex following TBI, and FB23-2 (an FTO inhibitor) contributes to the recovery of neural function in TBI. A study conducted in C56BL/6J mice revealed that m6A levels were elevated globally after transient focal ischemia, and the FTO level was also downregulated; further measurements of the m6A profile after focal ischemia with microarrays indicated that 95% of the transcripts were significantly hypermethylated (Chokkalla et al., 2019). Researchers have revealed that METTL3 is upregulated and FTO is downregulated in AD. More importantly, although some research has focused on the m6A modification in CNS injury disease, the downstream mechanism of these significantly changed genes with peaks has not been illustrated, and it awaits further investigation. There is currently no research that has focused on creating an m6A methylation atlas of TSCI in mammals. Our research fills this gap and provides potential genes that can be considered as key targets for TSCI treatment.

## Conclusion

This study demonstrates that the m6A modification and RNA expression levels change after TSCI, as determined by MeRIP-Seq. Some genes were identified in this study such as *Tlr4* and *Ngf* showed alteration in both expression and methylation level, providing substantial indicator in the epigenomic study of TSCI.

## Data Availability Statement

The datasets presented in this study can be found in online repositories. The names of the repository/repositories and accession number(s) can be found below: https://www.ncbi.nlm.nih.gov/, PRJNA791459.

## Ethics Statement

The animal study was reviewed and approved by Institutional Animal Care and Use Committee of Wuhan University (IACUC: ZN2021119).

## Author Contributions

YZ and XJ conceived and designed the research plans, supervised, and fixed the writing. ZZ, XZ, RL, and PW established the TSCI model and isolated RNA samples from cerebral cortex tissues. JY and HC completed the data processing, normalization, and bioinformatics analyses. JY and HC wrote the article with contributions from all the authors. All authors have read and approved the manuscript.

## Conflict of Interest

The authors declare that the research was conducted in the absence of any commercial or financial relationships that could be construed as a potential conflict of interest.

## Publisher’s Note

All claims expressed in this article are solely those of the authors and do not necessarily represent those of their affiliated organizations, or those of the publisher, the editors and the reviewers. Any product that may be evaluated in this article, or claim that may be made by its manufacturer, is not guaranteed or endorsed by the publisher.

## Footnotes

1
http://primer3.ut.ee/

## Abbreviations

TSCI: traumatic spinal cord injury
m6A: N6-methyladenosine
qRT-PCR: Quantitative reverse transcription polymerase chain reaction
MeRIP-qPCR: methylated RNA immunoprecipitation-qPCR
MeRIP-Seq: methylated RNA immunoprecipitation sequencing.
